# Evaluation of preparation methods for suspended nano-objects on substrates for dimensional measurements by atomic force microscopy

**DOI:** 10.3762/bjnano.8.179

**Published:** 2017-08-28

**Authors:** Petra Fiala, Daniel Göhler, Benno Wessely, Michael Stintz, Giovanni Mattia Lazzerini, Andrew Yacoot

**Affiliations:** 1 Research Group Mechanical Process Engineering, Institute of Process Engineering and Environmental Technology, Technische Universität Dresden, Münchner Platz 3, D-01062 Dresden, Germany; 2 National Physical Laboratory, Hampton Road, Teddington, Middlesex, TW11 0LW, UK

**Keywords:** atomic force microscope, nano-object, particle preparation

## Abstract

Dimensional measurements on nano-objects by atomic force microscopy (AFM) require samples of safely fixed and well individualized particles with a suitable surface-specific particle number on flat and clean substrates. Several known and proven particle preparation methods, i.e., membrane filtration, drying, rinsing, dip coating as well as electrostatic and thermal precipitation, were performed by means of scanning electron microscopy to examine their suitability for preparing samples for dimensional AFM measurements. Different suspensions of nano-objects (with varying material, size and shape) stabilized in aqueous solutions were prepared therefore on different flat substrates. The drop-drying method was found to be the most suitable one for the analysed suspensions, because it does not require expensive dedicated equipment and led to a uniform local distribution of individualized nano-objects. Traceable AFM measurements based on Si and SiO_2_ coated substrates confirmed the suitability of this technique.

## Introduction

Today, nanotechnology is an increasingly important part of particle technology, owing to the diversity of applications of nanoparticles (NP) in product improvement and development especially in food industry [1], pharmacy, cosmetics and medicine [2].

According to ISO/TS 80004-1:2010 [3], nanomaterials can be classified either as nanostructured materials; internal dimension ≤100 nm, external dimension typically greater than 100 nm or as nano-objects, i.e., individualized particles with at least one dimension ≤100 nm. Another widely used nanomaterial definition is the recommendation of the European Commission [4]. The recommendation focuses on the number-weighted constituent particle size distribution by which a material is classified as nanomaterial when the median constituent particle diameter is ≤100 nm. This is seen, especially for complex-nanostructured particles, as a metrological challenge for particle measurement instruments other than imaging ones [5]. Interestingly, a comparison between several nanoparticle measurement instruments including imaging methods showed a relative deviation up to 100% between the determined number-weighted median diameters [6]. According to this study, imaging methods such as scanning electron microscopy (SEM), transmission electron microscopy (TEM) or atomic force microscopy (AFM) are seen as most appropriate methods for nanomaterial classification and therefore traceability for these methods will become more and more important [7].

Despite the strengths of these imaging methods for nanomaterial classification, the quality of the granulometric results depends strongly on the sample preparation performed prior the actual measurement. Dimensional measurements by AFM, SEM or TEM require the following sample properties:

- high purity of substrates
- sufficient fixation of nano-objects without use of adhesive agents
- homogeneous deposition of nano-objects over the whole substrate
- clearly defined and retrievable preparation areas
- adequate surface-specific particle number
- well separated or low clustered particles/aggregates
- no deposition of stabilizers and additives on the substrates

Today, there is a multitude of preparation methods for electron microscopy [8], but most of them have not been analysed systematically. Thus this study aimed to identify preferable preparation methods for depositing suspended nano-objects on substrates taking into account both particle/suspension and substrate properties (e.g., surface potential of material and substrate, tendency of nano-objects to agglomerate, adhesion force, size and shape). Therefore several preparation methods (membrane filtration, drying, rinsing, dip coating, electrostatic precipitation, thermal precipitation) on silicon (Si) and with silicon dioxide (SiO_2_) coated Si substrates were analysed in combination with different nano-objects in aqueous solution, which vary in chemical composition, sizes and shape.

## Experimental details

### Nanomaterials and substrates

Table 1 provides information on the aqueous suspensions of nano-objects, which were used in this study for the characterization of the preparation methods. The chosen nano-objects belong to the material group that was identified as important for risk assessment or as reference nanomaterials [9].

**Table 1:** Overview of analysed nanomaterials and their characteristics.

material, shape	nominal size	density	*c*_M_^a^	stabilizer	supplier
[–]	[nm]	[kg/m^3^]	[mg/ml]	[–]	[–]

Au, spherical	60	19000	0.05	sodium citrate 2 mM	NanoComposix
Au, rod	25 × 77	19000	0.06	water, <0.1% CTAB^b^	Nanopartz
Ag, spherical	50	10500	0.02	sodium citrate 2 mM	NanoComposix
Ag, wire	60 × 10000	10500	0.02	isopropyl alcohol	Sigma-Aldrich
SiO_2_, spherical	100	2350	10	silanol	NanoComposix
TiO_2_, fractal	<150^c^	4230	350	n/a	Sigma-Aldrich

^a^mass concentration, ^b^cetrimonium bromide (CTAB), ^c^primary particle size = 21 nm.

The preparations were performed by means of hydrophilic track etching membranes made of polycarbonate (pore size 30 nm and 50 nm, Sterlitech Corp, Kent, USA and 100 nm, Merck Millipore, Darmstadt, Germany) as well as on silicon wafers (crystal orientation <100>) and on silicon wafers (crystal orientation <100>) coated with silicon dioxide. SEM measurements were performed for all substrates, whereas only the Si wavers and the SiO_2_ coated wafers were used for AFM measurements.

### Measurement devices

#### SEM

A low voltage high resolution SEM (Modell Gemini 982, Carl Zeiss AG, Jena, Germany) was operated for deposition quality assessment. Line grids with a nominal distance of 700 nm (MOXTEK Inc., Orem, Utah, USA) were used for instrument calibration.

#### AFM

AFM measurements were performed with a traceable atomic force microscope that uses two integrated optical interferometry systems for detecting the deflection of the cantilever and for measuring the vertical motion of the piezoelectric transducer (PZT) while operating in closed loop [10]. The optical interferometer used for measuring the *z*-displacement is the National Physical Laboratory’s (NPL) Plane Mirror Differential Optical Interferometer (PMDOI) [11], a homodyne differential interferometer, fibre-fed with a He-Ne frequency stabilized laser (λ = 632.8 nm). Two parallel mirrors are required for the interferometer; one for each optical path. One mirror is rigidly connected to the PZT tube that moves the cantilever, and the other forms the sample holder. The interferometer is a double pass interferometer with each optical path having two reflections from the mirror in its path, making one fringe equivalent to a displacement of λ/4 (158 nm). Using the interference of the two optical paths, the PMDOI traceably measures the relative displacement between the tip and the sample. The traceability achieved as optical interferometry is the primary route to traceability for dimensional metrology, and in this case it is realized using a frequency-stabilized He-Ne laser [12]. This results in improved accuracy and traceability over commercial AFMs [11].

The measurements were carried out using the AFM in closed-loop, non-contact mode in a temperature-controlled environment (20 ± 0.01 °C). Nanosensors PPP-NCLR tips (i.e., point probe plus non-contact long cantilever reflex coating) with nominal tip radius <10 nm were used. The AFM images were numerically corrected for tilt using the “mean plane subtraction” and “correction of horizontal scars” tools in Gwyddion [13].

Under the assumption that the NP are spherical and that the nanorods (NR) have a circular section, after doing a plane fit, their diameter are extracted by measuring the height of the nano-objects by subtracting the maximum value of each NP or NR by the value of the zero-plane. This is because an AFM image is the dilation of the tested surface by the tip. As a result, while the height of an isolated particle or rod on the surface remains the same in the AFM image, its lateral dimensions become broader due to the finite dimension of the AFM tip. Therefore, for an accurate measurement of the particle size, the height measurement should be used.

### Preparation methods

Particle preparation methods for analysis by electron microscope typically consist of three main stages:

- pre-conditioning of the initial sample (e.g., homogenisation, dilution) and the substrate (e.g., cleaning)
- application of conditioned sample on the conditioned substrate
- post-conditioning of sample/substrate (e.g., sputtering)

During the pre-conditioning of the sample each suspension was dispersed by ultrasonication (US bath, model SONOREX RK100, Bandelin electronic, Berlin, Germany) for between five and ten minutes and diluted with distilled and deionized water (ultrapure water with a specific electrical resistivity of 18.3 MΩ·cm) in the dependence of the known initial particle mass concentration and the given particle diameter (see Table 1) to reach *K*_C_-values (see Equation 1) between 2 and 10.

During the pre-conditioning of the substrates, the track etching membranes were rinsed with ultrapure water, and the silicon substrates were cleaned using either a wet chemical cleaning procedure or a dry cleaning procedure. The wet cleaning procedure was performed in accordance with the first step of the cleaning routine of the Radio Corporation of America (RCA) [14]. Thus, the substrate was immersed in a mixture of NH_4_OH (29%), H_2_O_2_ (30%) and deionized water in a volume ratio of 1:1:5 for 10 min at a temperature of 80 °C. In the case of the dry cleaning method, the plasma enhanced cleaning procedure (Plasma Prep II, SPI Supplies) was chosen. A study of cleaning methods for silicon has been undertaken in [15].

The application of conditioned samples on the conditioned substrates were performed by four liquid phase preparation methods and two gas phase preparation methods as summarised in Table 2. No additional post-conditioning of the sample/substrate was performed. For the SEM evaluation of membrane filtered samples it was helpful to sputter a thin (≈1 nm) platinum layer over the surface to prevent charging effects.

**Table 2:** Analysed preparation methods, their main deposition and transport mechanisms and corresponding references.

preparation method	deposition principle; transport mechanism	reference

Liquid phase preparation methods		

membrane filtration	sieve effect; convection to substrate	[16]
droplet drying	droplet application followed by evaporation	[17–18]
rinsing	immersion of substrate in suspension; diffusion to substrate	
dip coating	immersion of substrate in suspension; diffusion to substrate	[19–20]

Gas phase preparation methods		

electrostatic precipitation	electrostatic field forces	[21]
thermal precipitation	temperature gradient	[22]

#### Liquid phase preparation

As mentioned before, preparation should lead, beside a homogeneous particle distribution on the substrate surface, to an appropriate surface-specific particle number for subsequent size measurements, especially for AFM analyses.

A simplified approximation to estimate the surface-specific particle number *n*_A,PR_(*x*) on the substrate based on the particle number concentration *c*_N,PR_, the particle volume concentration *c*_V,PR_ or the particle mass concentration *c*_M,PR_ of the supplied suspension volume *V*_PR_, the wetted area *A*_PR_ and the particle size *x* is given in Equation 2, which relies on the assumption that spherically particles are individually and homogenously deposited over the whole substrate surface.

[2]
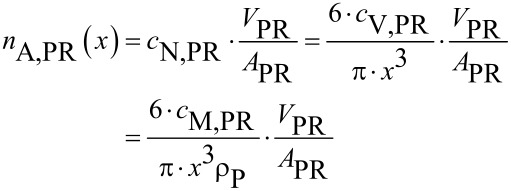


Considering the demand of individualized particles on the substrate, the loading of the substrate is limited based on the geometric dimensions of the particles and thus depends on the present particle size *x*. In the case of close-packing of equal spheres on the surface, the centre distance *D*_C_ between two neighbouring spheres corresponds to the diameter *x*. According to this, the distance should be at least ≥ *x*, so that the mean particle–particle-distance *D*_C_ can be expressed as the product of a multiple of *x*, here called *K*_C_ or dimensionless particle–particle-distance, and the particle size *x*.

[1]
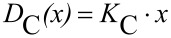


The surface-specific particle number *n*_A_(*x*) can also be expressed as a proportionality of the mean particle–particle-distance as given in Equation 3.

[3]
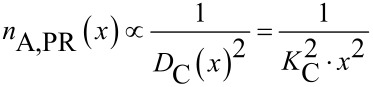


Note, that in the case of quadratic packing the proportional sign between *n*_A,PR_ and the term of Equation 3 converts into the equal sign. Based on experimental experience, it is recommended to achieve *K*_C_-values between 2 and 10 in order to achieve good results with regard to single-particle deposition.

**Figure 1:**
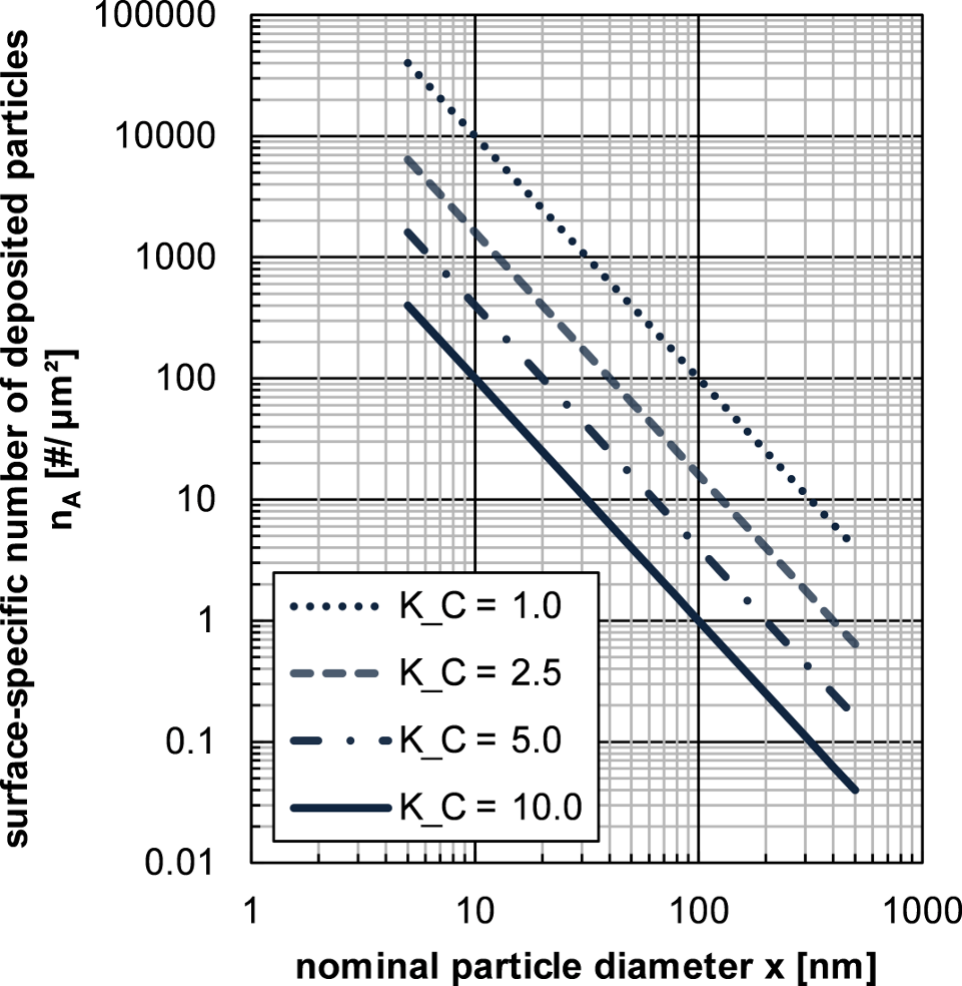
Theoretical surface-specific numbers of deposited particles (for quadratic packing) in dependence of the nominal particle diameter *x* and different *K*_C_-values.

Figure 1 shows the theoretical surface specific-numbers of deposited particles based on quadratic packing in the dependence of nominal particle diameter *x* and different *K*_C_-values. For orientation purposes, an image of 10 cm × 10 cm shows at a magnification of 1:10000 an surface area of 100 µm^2^, at a magnification of 1:100000 a surface area of 1 µm^2^.

This estimation is naturally applicable only for such preparation methods in which all of precipitated particles are deposited on the substrate, so the estimation is relevant for membrane filtration (if particle size > pore size) and droplet drying.

**Membrane filtration:** The membranes were fixed and flowed off in a filter element (stainless steel 316, diameter 13 mm, Merck Millipore, Darmstadt, Germany) and a suspension volume between 0.01–0.1 mL was dropped. The filtration was carried out with a vacuum pump producing an under pressure of 60 kPa. After membrane drying the filter unit was vented, the membrane carefully removed with tweezers and fixed on a specimen holder.

**Drying methods:** Conventional drop-drying was realized by supplying one droplet of the conditioned suspension (approx. 5–10 µL) on the conditioned substrate using a micropipette (Model L-200XLS+, Mettler Toledo, Gießen, Germany). The drying procedure was performed under ambient conditions within a laminar flow box (Model LF-VM-K0615; Steag Laminarflow Prozesstechnik GmbH, Pliezhausen, Germany) for at least 30 min (i.e., until complete drying). During the conventional drying procedure drying rings arise usually, where the nano-objects could agglomerate due to convective currents based on liquid evaporation [23]. Furthermore, stabilising agents in the suspension lead occasionally to salt residues on the surface which can disturb the analyses. Table 3 summarises test parameters for a suitable particle deposition on silicon wafers by conventional drop-drying.

**Table 3:** Particle concentration supplied by conventional drop-drying on Si wafers.

material, shape	nominal size	*c*_M,PR_	*V*_PR_	*A*_PR_	*c*_N_	*n*_A,PR_	*K*_C,PR_
[–]	[nm]	[mg/mL]	[µL]	[mm^2^]	[1/mL]	[1/µm^2^]	[–]

Au, sphere	60	0.025	8.7 ± 0.2	28 ± 4.6	1.16 × 10^10^	3.6	8.8
Au, rod	25 × 77^a^	0.015	8.7 ± 0.2	24 ± 4.3	1.2 × 10^10^	4.4	9.5
SiO_2_, spherical	100	0.014	8.7 ± 0.2	28 ± 4.6	1.14 × 10^10^	3.5	5.3
Ag, spherical	50	0.01	8.7 ± 0.2	24 ± 4.3	1.45 × 10^10^	5.3	8.7
Ag, wire	60 × 10000	0.25	8.7 ± 0.2	28 ± 4.6	n.a.	–	–
TiO_2_, fractal	<150^b^	0.025	8.7 ± 0.2	28 ± 4.6	2.2 × 10^10^	6.7	4.8

^a^Equivalent particle size 50 nm; ^b^equivalent particle size 80 nm.

The Marangoni-flow-assisted drop-drying method [18] prevents the formation of drying rings and lead therefore to a more uniform deposition of enhanced separated nanoparticles. Using this method, the drying procedure occurs in an ethanol vapour atmosphere, which was realized in this study in a petri dish. The thus induced gradient in surface tension causes the liquid to flow away from regions of low surface tension associated with a strong recirculation in the droplet, which removes particles from the contact line and moves them along the free surface toward the droplet centre.

**Rinsing method:** Apart from the interruption of drying after 5–10 min by rinsing with deionised water and a careful aspiration of the remaining liquid with a cleaning tissue, the rinsing method is similar to the one of simple drop-drying. This method reduces the salt residues on substrates but lead to a non-predictable surface specific particle number.

**Dip coating (Immersion):** In accordance with [20], the dip coating process consists of five stages. At first, the substrate is immersed in the conditioned suspension at a constant speed rate, where it rest for 10–15 min. A thin layer of the suspension adheres to the substrate after removal. The removal velocity therefore defines the layer thickness, i.e., an increase in the velocity leads to an increase in the thickness. Afterwards, excess liquid is drained and the remaining layer evaporated.

Dip coating is preferred method for producing (self-organising) thin layers on substrates, but particles also can be deposited individually in case of a corresponding initial concentration. The deposition rate can be set effectively and reproducibly via the residence time in the suspension.

#### Gas phase preparation

Over the last few decades several devices for the deposition of airborne particles on substrates have been developed and commercialized. The most important deposition mechanisms are the electrostatic precipitation [21], thermal precipitation [22,24–25] as well as aerosol filtration [26–27].

In contrast to the wet phase preparation methods where only the suspensions and substrates have to be pre-conditioned, gas phase preparation methods require two additional steps, i.e., aerosol generation (e.g., atomization) and aerosol conditioning (e.g., classifying, dilution, neutralisation). The most common method for this is the atomization of suspensions with subsequent aerosol drying (e.g., diffusion drying, convection drying). Both the generated droplet size and the state of dispersion (i.e., concentration and homogeneity) of the suspension influence the arising aerosol. Dissolved salts (e.g., stabilizers, trace elements) in the continuous phase of the suspension affect the particle size distribution due to crystallization during droplet drying. Thus, artificially generated aerosols are typically classified afterwards within differential electrical mobility classifiers according to ISO 15900:2009 [28]. To avoid an overlap of the nano-object mode and a residual mode in the particle size distribution, even finer droplets as generated by atomization are necessary as it can be realized by operating electrospray aerosol generators.

In this study, a two-component atomizer (Modell ATM 220, Topas GmbH, Dresden, Germany) and an electrospray aerosol generator (ESG, Modell 3480, TSI Inc. Shoreview, USA) were operated for aerosol generation. A differential mobility analyser (DMA, Model 3071, TSI Inc., USA) was used for aerosol classifying. Despite the considerable effort required for aerosol generation and conditioning, nanomaterial deposition from aerosols on substrates can be more accurately characterized than the deposition from suspensions, because available aerosol measurement systems (e.g., condensation particle counters, scanning mobility particle sizers) allow a high sensitively determination of both particle size and particle number concentration.

**Electrostatic precipitation:** During electrostatic precipitation, charged particles follow the lines of electric flux on the substrate within an electrical field. The deposition efficiency is considerably affected by the aerosol charge condition. Thus, electrostatic precipitators were occasionally equipped with ion sources, like corona needles for unipolar particle charging [21] or radionuclides for bipolar particle charging. Previous studies [29] have shown that electrostatic deposition can provide substrates with isolated particles if particle concentration, flow conditions and precipitation time are well balanced.

The electrostatic precipitation was performed using an electrostatic precipitator as developed by [21], which was operated with an aerosol sample flow rate of 0.3 L/min, unipolar particle charging by corona discharge at −3 kV and an electric field voltage of +13 kV. Details on the performed analyses are provided in Table 4.

**Table 4:** Analysed materials and details on aerosol generation for particle deposition by electrostatic precipitation.

material, shape	nominal size	atomised suspension	aerosol generation
[–]	[nm]	[–]	[–]

Au, spherical	60	2 mL stock suspension in 20 mL deionized water	atomisation by ATM 220, electrostatic classifying
Au, rod	25 × 77	75 vol % suspension + 25 vol % buffer solution^a^	atomisation by ESG 3480, no electrostatic classifying
SiO_2_, spherical	100	1 mL stock suspension in 60 mL deionized water	atomization by ATM 220, electrostatic classifying
TiO_2_, fractal	<150	0.2 mL stock suspension in 60 mL deionized water	atomization by ATM 220, electrostatic classifying

^a^500 mL deionized water + 0.77 g ammonium acetate + 0.75 mL 1 M ammonium hydroxide.

**Thermal precipitation:** The thermophoretic effect, which is the basis of the thermal precipitation technique, arises due to a temperature gradient between a warm and a cold plate. The more energetic molecule collisions from the warm side cause particle deposition on the cold plate - the substrate.

Within this study, a prototype of the personal thermal precipitator described in [22,24] was operated with an aerosol sample flow rate of 2.0 mL/min and a temperature difference of 15 K.

## Results and Discussion

### Sample preparation results

The major requirements for sample preparation are a pure uncontaminated substrate, realized by wet chemical procedures in case of silicon wafers, and a uniform deposition of particles sufficiently isolated from each other but with a concentration that allows meaningful measurements.

The following experimental specifications for preparation of isolated particles result from several empirical studies based on the K_C_ - theory (see section ‘Liquid phase preparation’) and were tested regarding their reproducibility as well as deposition quality and quantity. The characterisation by SEM shows examples of preparation results.

#### Liquid phase preparation results

**Figure 2:**
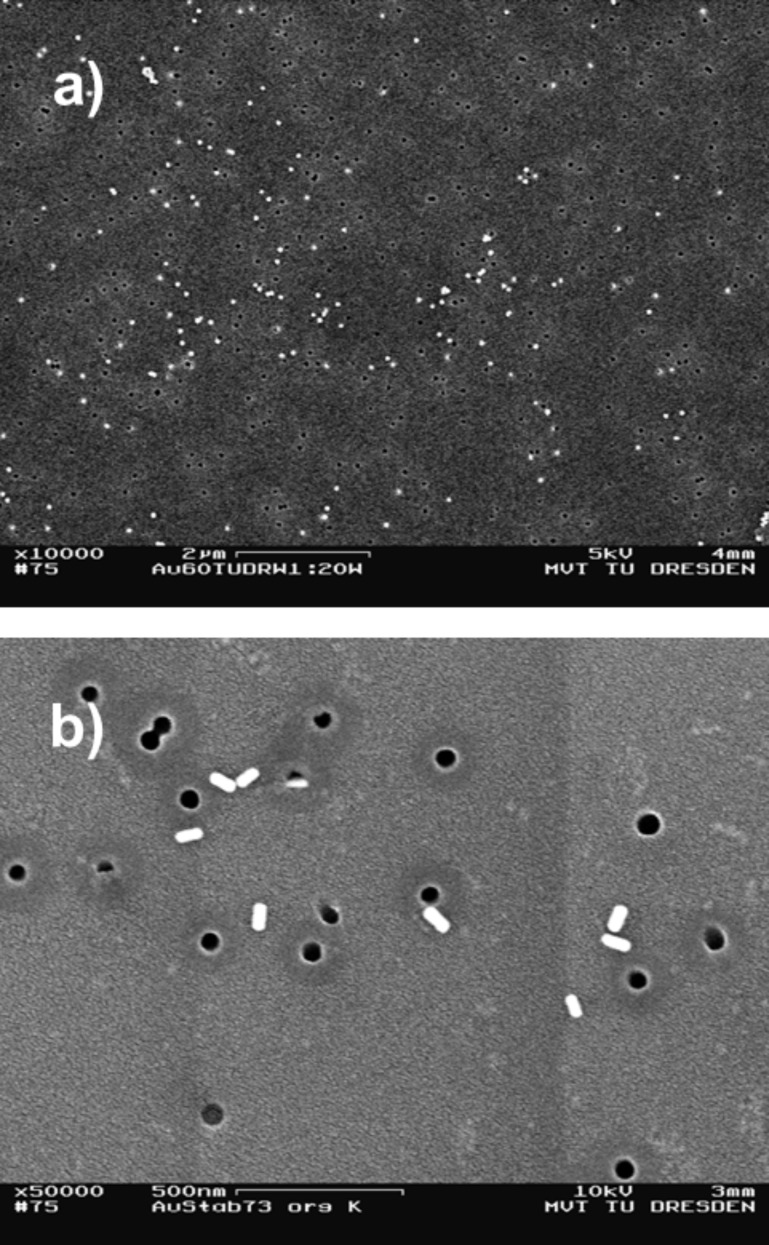
SEM images of a) spherical Au particles (60 nm) and b) Au rods (25 × 77 nm) prepared on track etching membranes by membrane filtration.

**Membrane filtration:** Preparations produced by membrane filtration allow the evaluation of a specific deposition rate; the number of particles contained in the relevant suspension volume could be compared with the total number of particles on the substrate. Earlier analysis showed, in the case of sufficient dilution of the suspension, a deposition of isolated particles on the membrane, as shown in Figure 2 for the gold particle systems. However, the roughness and the soft surface of the membrane substrate may adversely affect measurements by AFM, especially in case of higher cantilever forces.

**Figure 3:**
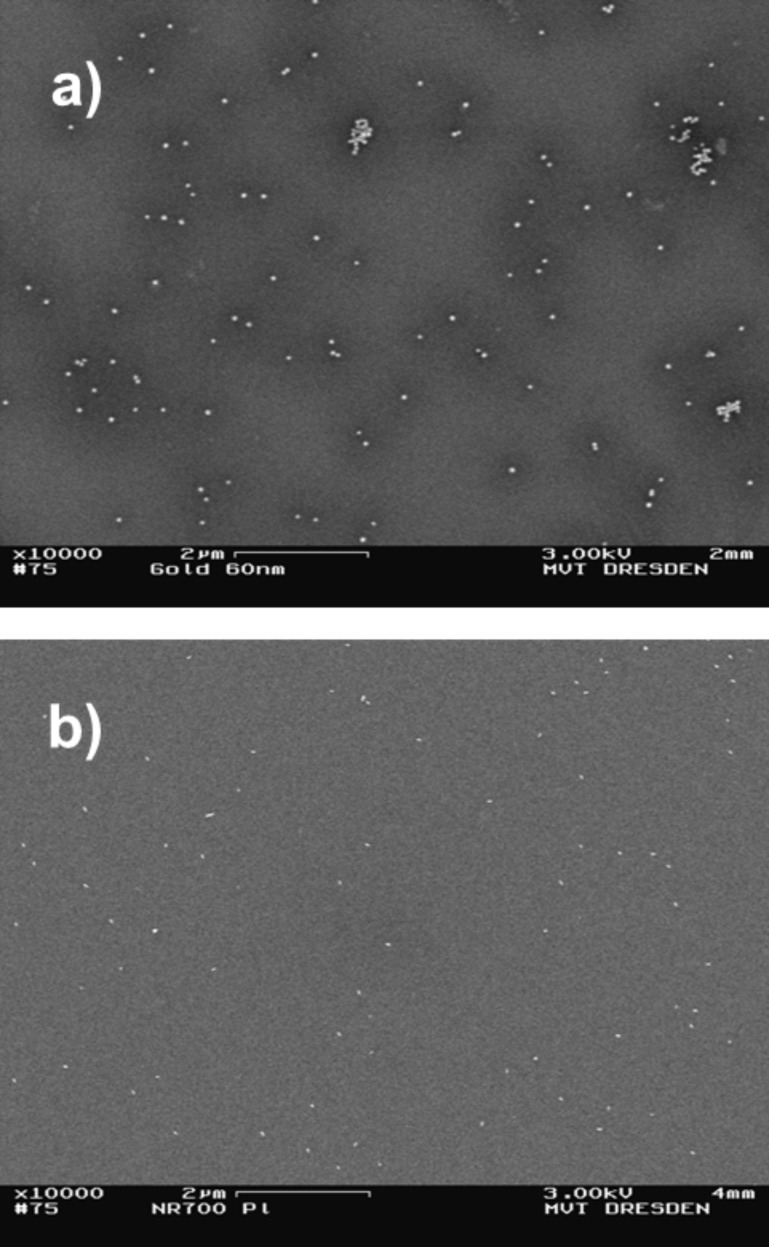
SEM images of a) spherical Au particles (60 nm) and b) Au rods (25 × 77 nm) prepared on Si wafers by conventional drop-drying.

**Figure 4:**
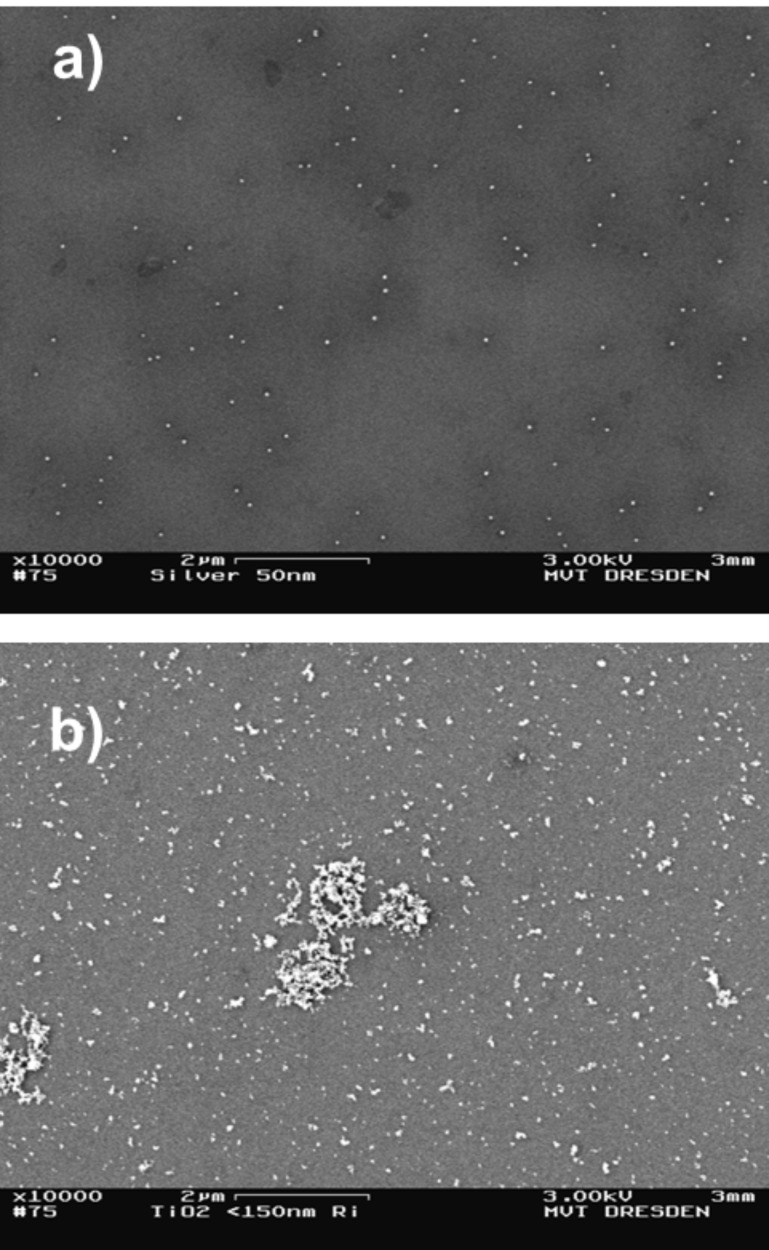
SEM images of a) spherical Ag particles (50 nm) and b) fractal TiO_2_ particles (<150 nm) prepared on Si wavers by conventional drop-drying.

**Drying methods:** Preparation by conventional drop-drying lead to moderate numbers of nano-objects per area adequately separated from each other on the substrate. On the one hand, there were numerous areas of predominantly isolated single particles beside a low number of agglomerated ones that could be well used for dimensional measurements by microscopy, see Figure 3 and Figure 4.

**Figure 5:**
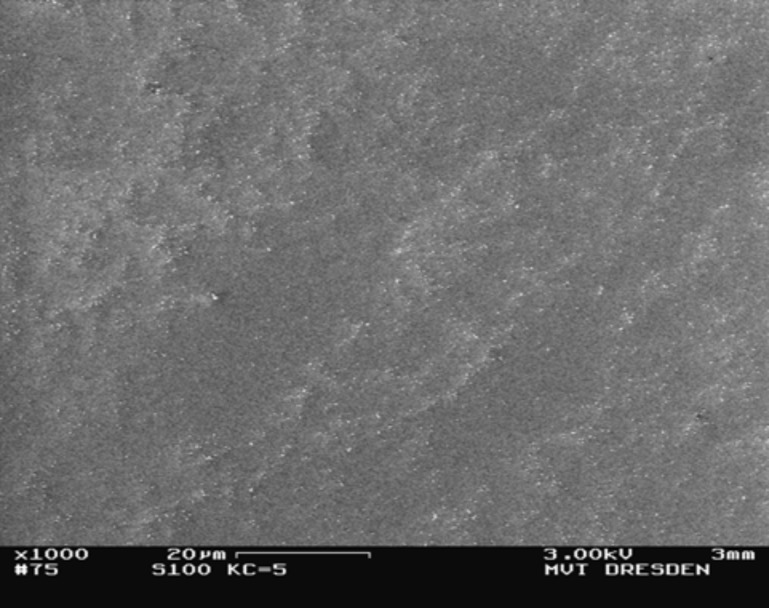
SEM overview image of spherical SiO_2_ particles (100 nm) prepared on Si waver by conventional drop-drying (*K*_C,PR_ ≈ 5) with characteristic non-homogeneous deposition structure.

On the other hand, poorer results were achieved regarding homogeneity of deposition caused by drying rings, surface effects, dispersion errors and sedimentation within the droplet. A SEM overview image for inhomogeneous deposited spherical SiO_2_ particles (100 nm) is shown in Figure 5.

**Figure 6:**
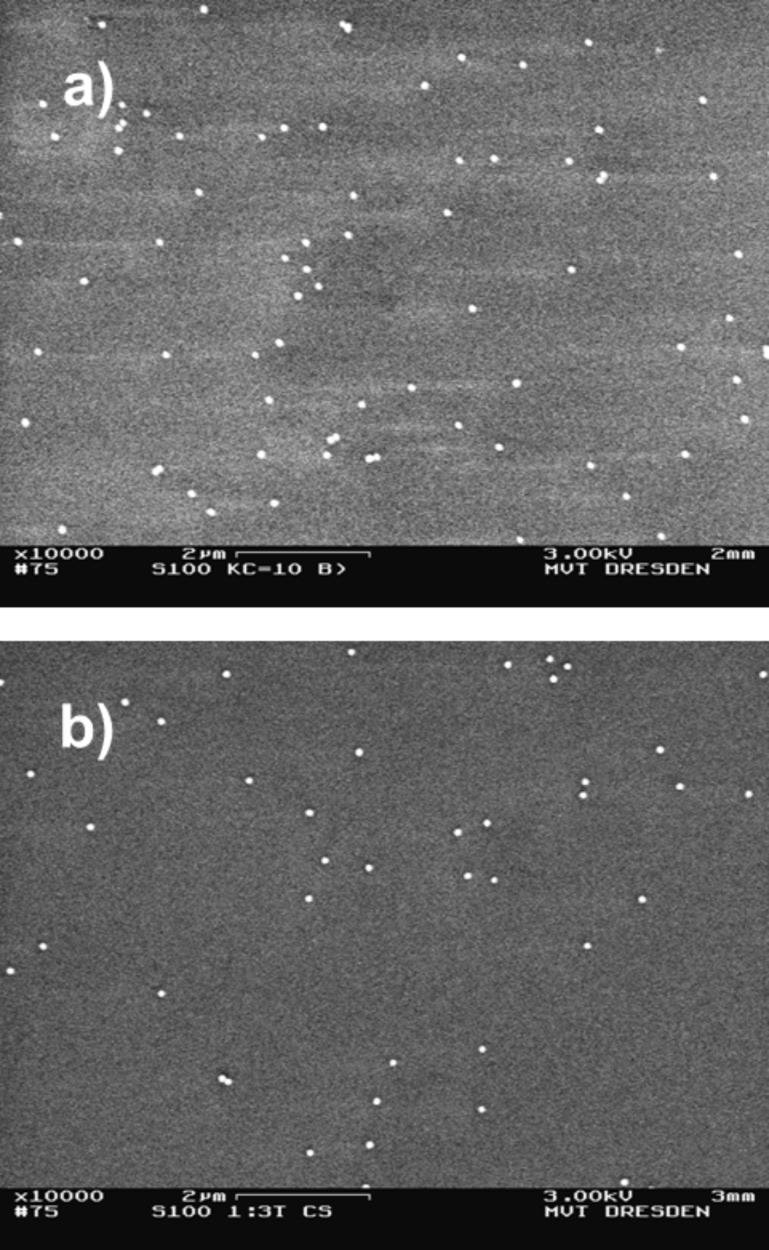
SEM images of spherical SiO_2_ particles (100 nm) prepared on Si wafers by a) conventional drop-drying (*K*_C,PR_ ≈ 10) and by b) Marangoni drop-drying (*K*_C,PR_ ≈ 10).

As it can be observed from Figure 6, the method of Marangoni-flow-assisted drop-drying produced excellent results. The determined use of the Marangoni effect enables the preparation of isolated particles, fewer agglomerates (doublets, triplets, …) and nearly stochastic homogeneously distributed particles on the substrate. The first and foremost reason for this is the intensive convection within the droplet during drying. Thus this preparation method is highly recommended for dimensional AFM measurements as well as for image based particle counting applications.

The drop-drying method enables an estimation of appropriate surface-specific particle number *n*_A,PR_ on the substrates. The investigations aimed to compare the real detectable particle number on the substrate with the theoretically calculated particle number using *K*_C._ The preparations were carried out with values of *K*_C,PR_ in the range of approximately 5 to 10.

Table 5 provides a quantitative comparison between the theoretical estimated surface-specific number of deposited particles *n*_A,PR_ with the experimental determined one *n*_A,exp_ for the analysed nanomaterials.

**Table 5:** Comparison between theoretical estimation and experimental determination of the surface-specific number of deposited particles based on conventional drop-drying.

material, shape	nominal size	*n*_A,PR_	*K*_C,PR_	*n*_A,exp_	*K*_C,exp_	*n*_A,exp_/*n*_A,PR_
[–]	[nm]	[1/µm^2^]	[–]	[1/µm^2^]	[–]	[–]

Au, spherical	60	3.6 ± 0.61	8.8 ± 0.74	0.8 ± 0.49	21.0 ± 8.84	0.22
Au, rod	25 × 77^a^	4.4 ± 0.79	9.5 ± 0.87	1.5 ± 0.02	16.2 ± 0.11	0.34
SiO_2_, spherical	100	3.5 ± 0.60	5.3 ± 0.45	3.3 ± 2.89	6.97 ± 3.00	0.94
Ag, spherical	50	5.3 ± 0.96	8.7 ± 0.79	2.5 ± 2.18	15.3 ± 5.86	0.47
TiO_2_, fractal	<150^b^	6.7 ± 1.15	4.8 ± 0.40	7.9 ± 6.96	5.3 ± 2.62	1.18

^a^Equivalent particle size 50 nm; ^b^equivalent particle size 80 nm.

The given relative standard deviation for *n*_A,exp_ is based on analyses of several hundreds of particles per material. The direct comparison between the mean values of *n*_A,exp_ and *n*_A,PR_ shows that the experimental values were mostly below 1, i.e., the samples contained fewer particles than theoretical predicted. The fractal TiO_2_ particles are an exception because of the difficulty in achieving their dispersion.

The surface-specific particle number depends on the characteristics of material and substrate, the type of stabilizers and the sample conditioning. All samples offered a good sticking behaviour, possibly because of the stabilizers used.

For the determination of the theoretical values of *n*_A,PR_, a constant particle concentrations were assumed. The main reason for the deviation between theoretical and experimental results of *n*_A_ and accordingly *K*_C_ we founded in the inhomogeneity of deposition. Further causes could be attributed to an insufficient sample homogenisation and dispersion, the drop-sampling, as different pipette drop volume or a remaining amount of particles in the pipette. We assumed an inaccuracy in the sampling volume of ±5 vol %. Another cause of the observed difference in drop dissemination was attributed to the different surface energy of the sample substrate. For the drop size measurement an inaccuracy of ±0.5 mm can be assumed.

In the case of oxides a good agreement between theoretical and experimental results of deposited mean particle numbers and thus of *K*_C_ could be observed, though the realisation of preparations with consistency was not possible. This leads to large standard deviations of the evaluated images. High deviations between theoretical and experimental results of deposited mean particle numbers were registered especially in case of metals. The reason for this is attributed to an incorrect number of particles in the preparation volume as a consequence of a low initial concentration. An availability of the materials in higher initial concentration would have increased the sampling quality.

#### Gas phase preparation results

**Figure 7:**
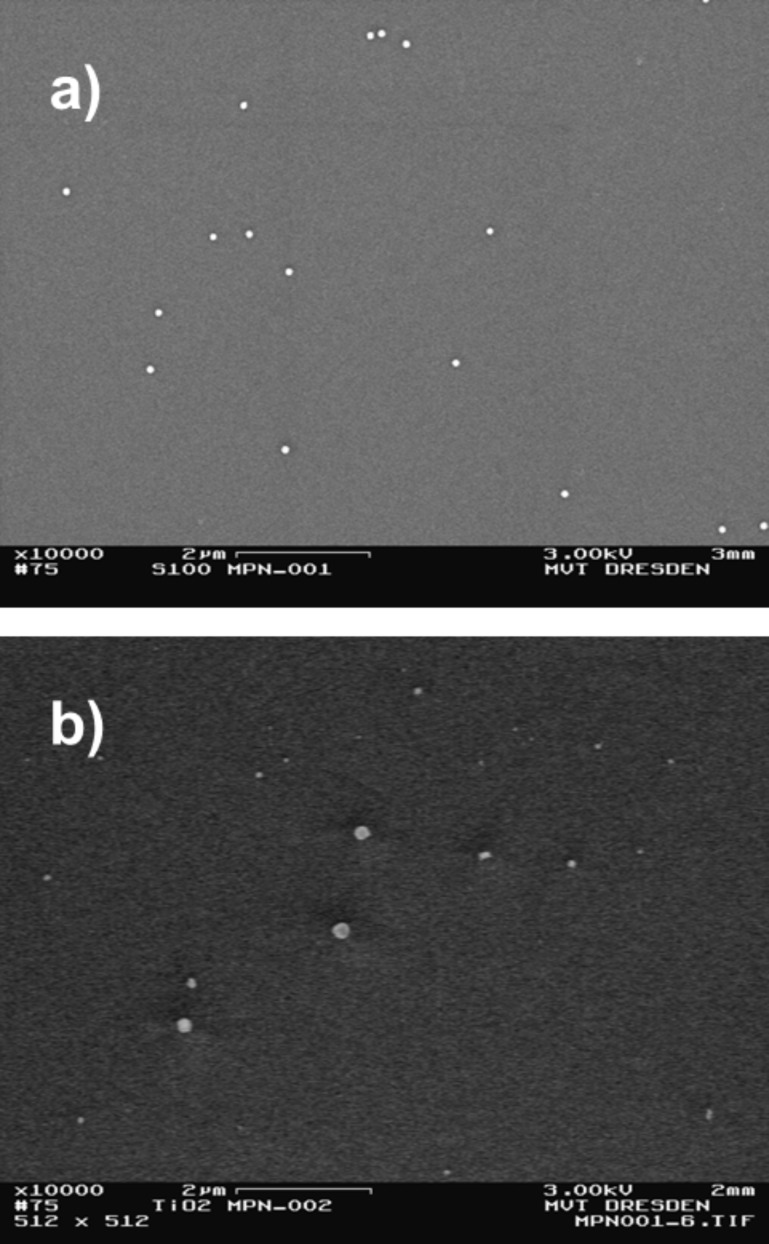
SEM images of spherical SiO_2_ particles (100 nm) and b) fractal TiO_2_ particles (<150 nm) prepared on Si wafers by electrostatic precipitation.

**Electrostatic precipitation:** Using the optimised initial concentrations for the aerosolization process as listed in Table 4, four materials were deposited on Si substrates. In the case of oxides, results showed an appropriate amount of particles, adequately separated from each other and well distributed over the substrate, shown in Figure 7. A radial symmetric deposition profile with an increased number of particles in the centre could be observed analogous to [21]. Using the example of SiO_2_ particles we captured a series of 14 SEM images and determined a mean *K*_C,exp_-value of 18. The pure metal particles (i.e., Au, Ag) could not be deposited sufficiently by electrostatic precipitation. This is attributed to the low supplier concentration and image charging effects.

**Thermal precipitation:** Preliminary analysis showed a deposition velocity of *v*_dep_(*x* = 60 nm) = 0.2 mm/s that was comparable to the one of 0.16 mm/s given in [24] that lead by supplying of an airborne particle number concentration of 8500 1/cm^3^ over a deposition time of 17.5 h to a surface-specific number of deposited particles of 0.038 1/µm^2^.

Thermal precipitation can provide substrates with isolated particles, but the low deposition velocity lead comparable high deposition time for suitable surface-specific particle numbers.

### Assessment of particle deposition quality

The preparation results are based on numerous experiments. The main investigations were carried out with SiO_2_ particles having a nominal 100 nm diameter; between 80 and 100 preparations were performed to identify the best reproducible conditions in terms of preparation method, particle concentration and type of substrate.

In the cases of Au- and Ag particles, rods and wires, analyses were focused on the different drying methods and dip-coating. To characterise the reproducibility, at least five repeated preparation procedures were performed for each sample material on the Si as well as on the SiO_2_-coated substrate.

The preparation quality of produced samples was evaluated from the point of view of cleanliness (in particular no deposition of stabilizers and additives on the substrate), and homogeneity of surface deposition with a satisfactory number of isolated particles. For subsequent dimensional measurements is it important to find such nano-objects homogeneously distributed over the substrate, at least in a clearly defined area and preferably without characteristic deposition profiles.

Table 6 gives an overview of preparation results of the selected nano-objects on track etching membranes (Membrane Filtration) and accordingly on Si and Si coated substrates (others) relating to their preparation quality grade.

**Table 6:** Assessment of preparation results concerning cleanliness, homogeneity of particle deposition, particle isolation and deposition quantity.^a^

material	preparation method	cleanliness	homogeneous deposition	particle isolation	surface-specific particle number

Au, spherical (60 nm)	mem. filtration drying dip coating rinsing el. precipitation	++ + + ++ −	+ + + − −	+ + + + +	+ + 0 0 −
Au, rod (25 × 77 nm)	mem. filtration drying dip coating rinsing el. precipitation	++ + + ++ +	++ ++ + ++ −	++ ++ + ++ +	+ + + + −
SiO_2_, spherical (100 nm)	mem. filtration drying dip coating rinsing el. precipitation	++ ++ ++ ++ ++	++ ++ + − ++	++ ++ ++ 0 ++	++ ++ + − ++
Ag, spherical (50 nm)	mem. filtration drying dip coating rinsing el. precipitation	++ + + ++ −	+ + 0 + −	++ + + + +	+ + + + −
Ag, wire (60 nm × 10 µm)	mem. filtration drying dip coating rinsing el. precipitation	++ ++ ++ ++ +	++ ++ ++ + −	0 0 0 0 +	++ ++ ++ + −
TiO_2_, fractal (<150 nm)	mem. filtration drying dip coating rinsing el. precipitation	++ + + ++ ++	++ + + ++ ++	0 0 0 + +	++ ++ + ++ ++

**^a^**++ very good, + good, 0 neutral, − bad.

### Results of AFM sharp tip measurements

**Figure 8:**
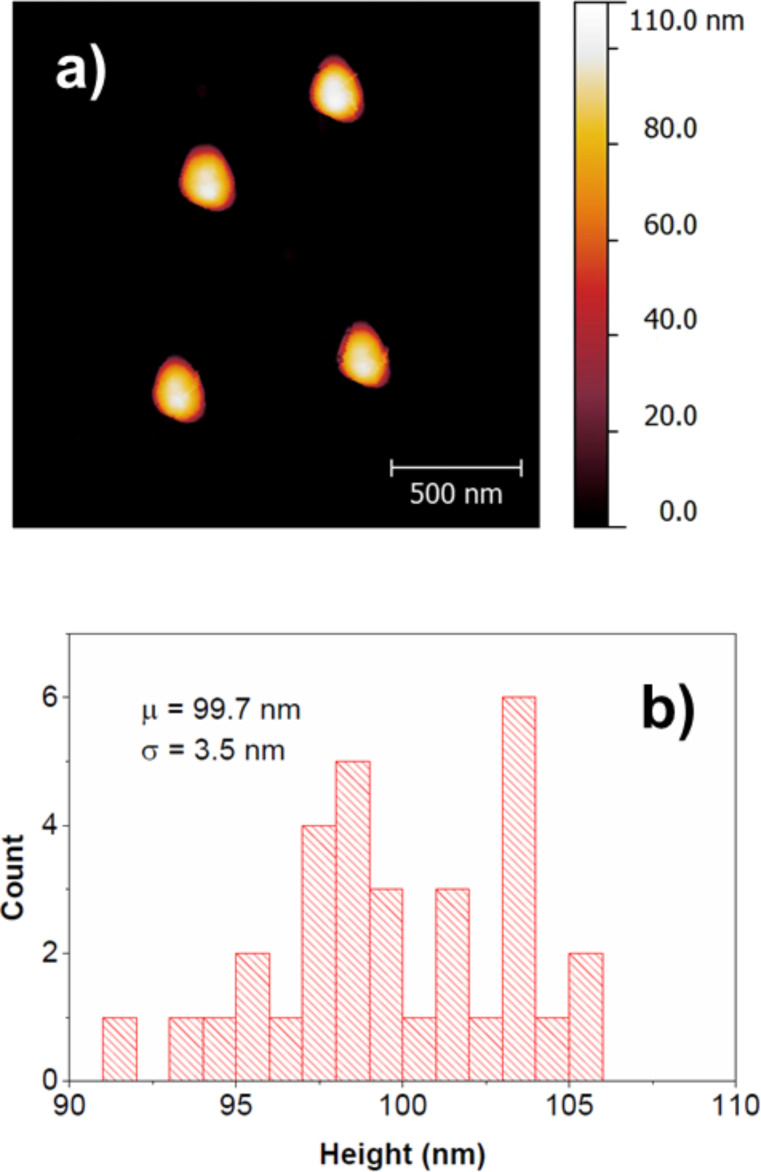
AFM analyses: a) AFM image and b) distribution of measured heights for spherical SiO_2_ particles (100 nm) deposited on Si waver.

Figure 8 provides exemplarily one AFM result concerning the height measurement of spherical SiO_2_ particles (100 nm) on Si, whereas Table 7 summarises all results.

**Table 7:** Results based on AFM height measurements.

material, shape	nominal size	substrate	*x*_AFM_	σ_SD_	*n*_P_
[–]	[nm]	[–]	[nm]	[nm]	[–]

TiO_2_, fractal	<150 nm	Si <100>	27.6	10.8	114
TiO_2_, fractal	<150 nm	SiO_2_	27.3	11.2	527
Au, rod	25	Si <100>	20.0	1.9	44
Au, rod	25	SiO_2_	21.3	2.2	56
Au, spherical	60	Si <100>	57.1	6.8	10
Au, spherical	60	SiO_2_	48.3	7.9	10
Ag, spherical	50	Si <100>	46.1	6.2	20
Ag, spherical	50	SiO_2_	50.4	8.7	33
SiO_2_, spherical	100	Si <100>	99.7	3.5	32

As it can be expected from Table 7, the average diameter determined by AFM can deviate quite significantly from the nominal size value. As an example the transverse diameter of 20.0 ± 1.9 nm of Au, rod on Si measure 20% less than the nominal size value of 25 nm. No significant difference in the determined average diameters could be observed between the two substrates.

## Conclusion

The investigations showed that conventional drop-drying is a suitable method to deposit nanoparticles and nanorods on Si and SiO_2_ substrates, which enables the performance of quantitative analyses by atomic force microscopy. The results showed the suitability of this preparation method which has the additional advantage of being easily carried out in any laboratory without additional specialist equipment. The drying in an ethanol vapour atmosphere is the most suitable technique; it allows a distribution of nano-objects much more uniformly and avoids the formation of agglomerates. It also was remarked that drying methods, in particular the conventional drop-drying, include uncertainties regarding the quantitative analysis caused by inhomogeneous deposition and sampling errors. In addition the sampling had to be optimised for better compliances between theoretical predicted and effectively prepared nano-objects.

Despite high device-related effort, the preparation by electrostatic precipitation also has yielded a homogeneous deposition of perfect isolated particles and thus appropriate for the sample preparation for microscopic measurements.

Using an atomic force microscope that realizes its traceability directly using an integrated optical interferometer we confirmed the suitability of the technique for producing samples whose dimensions can be measured accurately by AFM.
